# Engaging religious leaders to promote safe burial practices during the 2014–2016 Ebola virus disease outbreak, Sierra Leone

**DOI:** 10.2471/BLT.20.263202

**Published:** 2021-03-02

**Authors:** Padraig Lyons, Maike Winters, Zangin Zeebari, Kirsten Schmidt-Hellerau, Paul Sengeh, Mohammad B Jalloh, Mohamed F Jalloh, Helena Nordenstedt

**Affiliations:** a Department of Global Public Health, Karolinska Institutet, Tomtebodavägen 18B, 17165 Solna, Sweden.; b Department of Economics, Finance and Statistics, Jönköping International Business School, Jönköping Sweden.; c FOCUS1000, Freetown, Sierra Leone.; d Division of Global Health Protection, US Centers for Disease Control and Prevention, Atlanta, United States of America.

## Abstract

**Objective:**

To quantify the potential impact of engaging religious leaders in promoting safe burial practices during the 2014–2016 Ebola virus disease outbreak in Sierra Leone.

**Methods:**

We analysed population-based household survey data from 3540 respondents collected around the peak of the outbreak in Sierra Leone, December 2014. Respondents were asked if in the past month they had heard an imam or pastor say that people should not touch or wash a dead body. We used multilevel logistic regression modelling to examine if exposure to religious leaders’ messages was associated with protective burial intentions if a family member died at home and other Ebola protective behaviours.

**Findings:**

Of the respondents, 3148 (89%) had been exposed to faith-based messages from religious leaders on safe Ebola burials and 369 (10%) were unexposed. Exposure to religious leaders’ messages was associated with a nearly twofold increase in the intention to accept safe alternatives to traditional burials and the intention to wait ≥ 2 days for burial teams (adjusted odds ratio, aOR: 1.69; 95% confidence interval, CI: 1.23–2.31 and aOR: 1.84; 95% CI: 1.38–2.44, respectively). Exposure to messages from religious leaders was also associated with avoidance of traditional burials and of contact with suspected Ebola patients (aOR: 1.46; 95% CI: 1.14–1.89 and aOR: 1.65; 95% CI: 1.27–2.13, respectively).

**Conclusion:**

Public health messages promoted by religious leaders may have influenced safe burial behaviours during the Ebola outbreak in Sierra Leone. Engagement of religious leaders in risk communication should be prioritized during health emergencies in similar settings.

## Résumé

**Objectif:**

Quantifier l'impact potentiel de l'implication des autorités religieuses dans la promotion de pratiques funéraires sûres durant l'épidémie de maladie à virus Ebola entre 2014 et 2016 en Sierra Leone.

**Méthodes:**

Nous avons analysé les réponses fournies par 3540 ménages ayant pris part à des enquêtes menées au plus fort de l'épidémie en Sierra Leone, en décembre 2014. Il avait été demandé aux participants si, au cours du mois écoulé, ils avaient entendu un imam, un prêtre ou un pasteur dire que les gens ne devaient pas toucher ni laver le corps d'un défunt. Nous avons employé un modèle de régression logistique multiniveaux pour déterminer si l'exposition aux messages des autorités religieuses entraînait l'adoption de pratiques funéraires préventives en cas de décès d'un proche à domicile, et d'autres comportements de protection contre Ebola en général.

**Résultats:**

Sur l'ensemble des participants, 3148 (89%) avaient été exposés à des messages de la part de leurs chefs religieux concernant les pratiques funéraires limitant la propagation d'Ebola, tandis que 369 (10%) n'ont reçu aucune information à ce sujet. Ceux exposés aux messages des autorités religieuses ont été presque deux fois plus nombreux à accepter les alternatives aux enterrements traditionnels et à prévoir d'attendre ≥ 2 jours avant de faire appel aux équipes d'inhumation (odds ratio ajusté, ORA: 1,69; intervalle de confiance de 95%, IC: 1,23-2,31 et ORA: 1,84; IC de 95%: 1,38-2,44 respectivement). L'exposition aux messages de la part des chefs religieux a également permis d'éviter l'organisation de funérailles traditionnelles et le contact avec des patients susceptibles d'avoir contracté la maladie à virus Ebola (ORA: 1,46; IC de 95%: 1,14-1,89 et ORA: 1,65; IC de 95%: 1,27-2,13 respectivement).

**Conclusion:**

Le soutien des autorités religieuses aux messages de santé publique pourrait avoir eu une influence positive sur les comportements funéraires durant l'épidémie d'Ebola en Sierra Leone. Il faut privilégier l'implication des instances dirigeantes de chaque confession dans la communication des risques lorsque des urgences sanitaires surviennent dans des environnements similaires.

## Resumen

**Objetivo:**

Cuantificar el impacto potencial de la implicación de los líderes religiosos en la promoción de prácticas funerarias seguras durante la epidemia del ébola de 2014 a 2016 en Sierra Leona.

**Métodos:**

Analizamos las respuestas de 3.540 hogares que participaron en las encuestas realizadas en el momento álgido de la epidemia en Sierra Leona, en diciembre de 2014. Se preguntó a los participantes si, en el último mes, habían oído a un imán, sacerdote o pastor decir que la gente no debía tocar o lavar el cuerpo de un difunto. Se utilizó un modelo de regresión logística multinivel para determinar si la exposición a los mensajes de los líderes religiosos conducía a prácticas funerarias preventivas en caso de muerte de un familiar en el hogar, y a otros comportamientos de protección contra el ébola en general.

**Resultados:**

De todos los participantes, 3148 (89%) habían recibido mensajes de sus líderes religiosos sobre las prácticas funerarias que limitan la propagación del ébola, mientras que 369 (10%) no habían recibido ninguna información sobre el tema. Las personas expuestas a los mensajes de los líderes religiosos tenían casi el doble de probabilidades de aceptar alternativas a los entierros tradicionales y de planear esperar ≥ 2 días antes de llamar a los servicios funerarios (razón de probabilidades ajustada, aOR: 1,69; intervalo de confianza del 95%, CI: 1,23-2,31 y aOR: 1,84; CI del 95%: 1,38-2,44 respectivamente). La exposición a los mensajes de los líderes religiosos también evitó los funerales tradicionales y el contacto con pacientes con riesgo de contraer la enfermedad del ébola (aOR: 1,46; IC del 95%: 1,14-1,89 y aOR: 1,65; IC del 95%: 1,27-2,13 respectivamente).

**Conclusión:**

El apoyo de los líderes religiosos a través de los mensajes de salud pública puede haber influido positivamente en el comportamiento funerario durante la epidemia del ébola en Sierra Leona. La implicación de los líderes religiosos en la comunicación de riesgos debería ser prioritaria durante las emergencias sanitarias en entornos similares.

## ملخص

الغرض قياس الأثر المحتمل لإشراك الزعماء الدينيين في الترويج لممارسات الدفن الآمن أثناء الفترة من 2014 إلى 2016 خلال تفشي مرض فيروس أيبولا في سيراليون.

الطريقة قمنا بتحليل بيانات لمسح شمل أسر من السكان من 3540 مشاركًا، تم جمعهم من حول بؤرة تفشي المرض في سيراليون، وذلك في ديسمبر/كانون أول 2014. تم سؤال المشاركين عما إذا كانوا قد سمعوا في الشهر الماضي إمامًا أو قسًا يقول إنه لا ينبغي على الناس لمس جثة المتوفى أو تغسيلها. لقد استخدمنا أسلوبًا لوضع نماذج التحوّف اللوجستي متعدد المستويات للتحقق مما إذا كان وصول رسائل الزعماء الدينيين مرتبطًا بنوايا الدفن الوقائية في حالة وفاة أحد أفراد الأسرة في المنزل، وسلوكيات وقائية أخرى خاصة بإيبولا.

النتائج وصل لعدد 3148 مشاركًا (89%) رسائل إيمانية من الزعماء الدينيين بخصوص شعائر الدفن الآمن في فترة أيبولا، بينما لم يصل لعدد 369 مشاركًا (10%) هذه الرسائل. ارتبط التعرض لرسائل الزعماء الدينيين بزيادة مضاعفة تقريبًا في نية قبول البدائل الآمنة لطقوس الدفن التقليدية، وزيادة في نية الانتظار تزيد عن أو تساوي يومين لفرق الدفن (نسبة الاحتمالات المعدلة: 1.69؛ فاصل الثقة 95%: 1.23 إلى 2.31 ونسبة الاحتمالات المعدّلة: 1.84؛ ‏بفاصل ثقة 95%: 1.38 إلى 2.44، على التوالي). ارتبط التعرض للرسائل من الزعماء الدينيين أيضًا بتجنب طقوس الدفن التقليدي، والاتصال بمرضى إيبولا المشتبه بهم (نسبة الاحتمالات المعدّلة: 1.46؛ ‏بفاصل ثقة 95%: 1.14 إلى 1.89 ونسبة الاحتمالات المعدّلة: 1.65؛ ‏بفاصل ثقة 95%: 1.27 إلى 0.95، على التوالي).

الاستنتاج ربما أثرت رسائل الصحة العامة التي روج لها الزعماء الدينيون على سلوكيات الدفن الآمنة أثناء تفشي فيروس إيبولا في سيراليون. يجب منح الأولوية لإشراك الزعماء الدينيين في الإعلان عن المخاطر أثناء حالات الطوارئ الصحية في بيئات مماثلة. ****

## 摘要

**目的 旨在:**

量化 2014-2016 年塞拉利昂埃博拉病毒病爆发期间，让宗教领袖参与推广安全埋葬做法的潜在影响。

**方法:**

2014 年 12 月，我们在塞拉利昂埃博拉病毒病爆发高峰期收集了 3540 名受访者的人口家庭调查数据，并对这些数据进行了分析。我们询问受访者，在过去一个月中，他们是否曾听伊玛目或牧师说过不要触摸或清洗尸体。我们使用多层逻辑回归模型来检测，接触宗教领袖的信息是否与患者死于家中时家属的保护性埋葬意愿及其他埃博拉保护行为有关。

**结果:**

调查结果显示，3148 名受访者 (89%) 接触过宗教领袖传播的基于信仰的安全埋葬埃博拉患者尸体相关信息，而另外 369 名受访者 (10%) 并未接触过此类信息。接触宗教领袖的信息与愿意接受以安全做法代替传统埋葬方法并且愿意等待2 天排队埋葬的人数增加了近两倍（分别为，调整后优势比，aOR：1.69；95% 置信区间，CI：1.23-2.31，和 aOR：1.84；95% CI：1.38-2.44）有关。接触宗教领袖的信息还与避免采用传统埋葬方法和避免接触埃博拉疑似患者（分别为，aOR：1.46；95% CI：1.14-1.89，和 aOR：1.65；95% CI：1.27-2.13）有关。

**结论:**

塞拉利昂埃博拉病毒爆发期间，宗教领袖宣传的公共卫生信息可能对安全埋葬行为产生了积极影响。在发生类似卫生紧急情况时，应优先考虑让宗教领袖参与风险沟通。

## Резюме

**Цель:**

Количественная оценка потенциального влияния привлечения религиозных лидеров к пропаганде методов безопасного захоронения во время вспышки болезни, вызванной вирусом Эбола, в Сьерра-Леоне в 2014–2016 годах.

**Методы:**

Авторы проанализировали данные опросов населения по домохозяйствам от 3540 респондентов, собранные примерно на пике вспышки в Сьерра-Леоне в декабре 2014 года. Респондентам задавали вопрос о том, слышали ли они в течение последнего месяца от пастора или имама слова, что люди не должны трогать или мыть тело умершего. Было использовано многоуровневое моделирование логистической регрессии, чтобы изучить, связано ли воздействие посланий религиозных лидеров с защитными намерениями в отношении захоронения, если член семьи умер дома, и с другими видами защитного поведения от Эболы.

**Результаты:**

Из респондентов 3148 человек (89%) слышали религиозные послания от религиозных лидеров о безопасных захоронениях в связи с вирусом Эбола, а 369 человек (10%) — не слышали. Получение такой информации от религиозных лидеров было связано с почти двукратным увеличением намерения принять безопасные альтернативы традиционным захоронениям и с намерением ожидать прибытия бригад по захоронению в течение двух дней и более (скорректированное отношение шансов, сОШ: 1,69; 95%-й ДИ: 1,23–2,31 и сОШ: 1,84; 95%-й ДИ: 1,38–2,44, соответственно). Получение такого рода информации от религиозных лидеров также было связано с целью избежать традиционных захоронений и контактов с предполагаемыми пациентами с Эболой (сОШ: 1,46; 95%-й ДИ: 1,14–1,89 и сОШ: 1,65; 95%-й ДИ: 1,27–2,13, соответственно).

**Вывод:**

Послания в области общественного здравоохранения, распространяемые религиозными лидерами, могли повлиять на выбор более безопасного поведения в случае захоронения во время вспышки Эболы в Сьерра-Леоне. Привлечение религиозных лидеров к информированию о рисках должно быть приоритетным во время чрезвычайных ситуаций в области здравоохранения в аналогичных условиях.

## Introduction

The Ebola virus disease outbreak in West Africa between 2013 and 2016 is the largest in recorded history.^1^^,^^2^ Sierra Leone was heavily affected by the outbreak with over 14 000 suspected and confirmed cases.^2^ Because this outbreak was the first known Ebola outbreak in the region, knowledge of the disease in Sierra Leone was limited in the initial stages of the epidemic.^3^ Widespread public misconceptions and denial of the disease’s existence posed serious challenges to outbreak control efforts.^4^

Ebola is a zoonotic infection that is transmitted through person-to-person contact with infectious bodily fluids during an outbreak.^5^ The viral load in the host’s body is highest around the time of death, making the deceased Ebola victim highly infectious.^5^ In several West African countries, including Sierra Leone, washing (or ablution) and dressing of a deceased family member in preparation for burial are important religious and cultural traditions.^6^ Religion plays an important role in the lives of Sierra Leoneans, with the majority of Sierra Leoneans affiliating with either Islam or Christianity.^7^ As a result, religious traditions are seen as an essential part of life and death for many of the people. The handling of a deceased family member’s body, including their contaminated clothing and personal items, greatly increased the risk of infection spread. Traditional burial practices were considered major sources of Ebola transmission during the outbreak.^8^ Researchers have estimated that 2.5 new cases of Ebola resulted from every unsafe burial.^9^ Numerous case clusters were epidemiologically linked back to traditional burial ceremonies, including the index case in Sierra Leone.^10^^,^^11^

Because of their influential role in Sierra Leone, the Religious Leaders’ Task Force on Ebola was established in July 2014 to enhance the role of faith-based organizations in the Ebola response.^12^ Religious leaders had traditionally promoted various forms of physical contact with corpses including washing of the deceased as part of long-standing funeral and burial rituals.^13^^,^^14^ Therefore, a key intervention to improve the uptake of safe burials was to engage religious leaders to promote new messages to counteract their pre-Ebola messages.

In early October 2014, the World Health Organization (WHO) published updated protocols for the safe and dignified burial of corpses during the outbreak.^15^ By this time, there were over 2000 confirmed Ebola cases in Sierra Leone.^16^ As per WHO guidelines, specialized burial teams were established by WHO to handle burials in a safe, supervised and controlled manner – irrespective of the suspected cause of death.^15^ Safe and dignified burial protocols eventually incorporated feedback from religious leaders who provided guidelines for culturally appropriate modifications to traditional burials.^15^^,^^17^ Burial teams were trained to respect the spiritual requirements of Sierra Leoneans by providing safe alternatives such as performing dry ablution and shrouding of the deceased, allowing families to observe the burial and having a religious leader say a customary final prayer.^18^^,^^19^ In addition to burial teams, a national call centre was established by the Sierra Leonean government for communities to report deaths of all causes during the outbreak.^20^ Government policy at this time directed that burial teams should respond to all reported deaths within 24 hours.^17^ However, burial team capacity was not always able to meet high community demand, particularly within this narrow time frame, with up to 3000 death alerts received in a single day in December 2014.^20^ Consequently, burial teams took up to 2 or more days to respond to some death notifications.^20^

In times of crisis, people will turn to those they have trusted in the past for information.^21^ Religious leaders are trusted figures in Sierra Leonean society and were recognized as influential spokespersons for risk communication campaigns during the outbreak.^7^^,^^21^ Over 6000 religious leaders countrywide were trained on the promotion of faith-based Ebola messages between October and December 2014 by the Social Mobilization Action Consortium, which included local and international organizations in support of the Sierra Leone Ministry of Health and Sanitation.^22^^,23^ In collaboration with public health professionals, religious leaders developed faith-based messages that were delivered nationwide in places of worship and local community events as well as through radio and television programmes.^12^^,^^24^ Religious leaders facilitated trust by engaging the public in dialogue around accurate measures of Ebola prevention, while attempting to address people’s concerns and helping to dispel Ebola misinformation.^12^ The messages covered key aspects of Ebola risk communication, drawing upon scriptural texts to promote safe avoidance of physical contact with deceased household members and appealing to people to make use of specialized burial teams.^25^

It has been speculated that religious leaders’ involvement in burial teams played an important role in creating an enabling social environment for the uptake of safe burial practices during the outbreak in West Africa.^26^ Empirical data on the impact of engaging religious leaders in promoting protective Ebola behaviours have not been documented in the published literature to date. We therefore aimed to examine the effects of public health messages delivered by religious leaders on safe burial intentions and protective behaviours regarding Ebola during the outbreak in Sierra Leone.

## Methods

### Study setting and sample

We obtained population-based data from a cross-sectional household knowledge, attitudes and practices survey from the 2014–2016 Ebola outbreak in Sierra Leone conducted by the Sierra Leone National Social Mobilisation Pillar with support from local and international organizations.^3^ The survey was conducted in all 14 districts of the country over a 2-week period in December 2014, just after the peak of new cases of Ebola in Sierra Leone.^3^^,^^27^ Sample size calculations estimated 800 households and 3600 respondents to produce regional-level estimates of key Ebola protective measures. The methods of the survey have been described in detail elsewhere.^3^^,^^8^

Multistage cluster sampling was used to randomly select enumeration areas (clusters) from the country’s 2004 census sampling frame. Within each cluster, households were randomly selected, followed by the selection of two participants from each household. The head of the household was always selected in addition to a second random household member who was either an adult woman (aged 25 years or older) or a young person (aged 15–24 years). Consent was obtained from a parent or guardian before any child’s involvement in addition to obtaining assent directly from the child. 

### Variables selected

Our main independent variable was respondents’ exposure to religious leaders’ messages promoting safe Ebola burials. This information was obtained in the survey using the question: *“*In the past month, have you heard an imam/pastor say that during this period people should NOT touch or wash a dead body?” Other independent variables we selected for analysis were the demographic characteristics of respondents: region of residence, age, sex, educational level and religion.

We selected five outcome variables for analysis. Three outcome variables reflected behavioural intentions if a family member died at home: (i) intending to accept safe alternatives to traditional burials; (ii) intending to avoid touching or washing the corpse; and (iii) intending to wait 2 or more days for a burial team to arrive. Two outcome variables reflected protective behaviours against Ebola transmission: (iv) avoiding unsafe traditional burials and (v) avoiding physical contact with suspected Ebola patients. We selected these five outcome variables because they closely reflected key aspects of attitudes and behaviour that were promoted in the faith messages delivered by religious leaders. 

Further descriptions of the independent and outcome variables we selected for inclusion in our analyses are provided in the data repository.^25^

### Statistical analysis

We generated unweighted frequencies and proportions for all independent and outcome variables. We conducted multilevel logistic regression modelling to account for the intraclass correlation among respondents from the same geographical cluster. We used multilevel logistic regression models to examine the association between exposure to religious leaders’ messages and the three binary outcomes on safe burial intentions and the two binary outcomes on protective behaviours against Ebola transmission. In addition, we fitted two multilevel logistic regression models to further investigate the association between intention to wait for a burial team for 2 or more days and respondents’ protective behaviours. All multilevel logistic regression models were adjusted for respondents’ region of residence (west, south, east, north), age (15–24 years, ≥ 25 years), sex (male, female), religion (Islam, Christianity) and educational level (no formal education, some primary, secondary and above). We performed statistical analysis using Stata statistical software, version 16 (StataCorp LLC, College Station, United States of America).

### Ethical approval

We obtained ethical approval for our study from Sierra Leone Research and Scientific Review Committee. The Center for Global Health at the United States Centers for Disease Control and Prevention determined that the assessment was part of the public health response to the Ebola outbreak in Sierra Leone, and was determined to be non-research. The secondary data analysis protocol was further approved by the ethical review board at Karolinska Institutet, Stockholm, Sweden (DNR 2018/1276–31).

## Results

A total of 3540 respondents participated in the survey (98% of the 3612 people who were asked to participate). One third of the respondents (1177; 33%) were younger than 25 years, with an approximately equal distribution of males (1809; 51%) and females (1731; 49%; Table 1). About a third of respondents (1194; 34%) had no formal education. Attainment of secondary education or higher was more commonly reported among respondents who resided in the western region, where the capital city Freetown is situated (562/812; 69%).

**Table 1:** Demographic characteristics and reported behaviours of survey respondents, by exposure to messages on safe burials from religious leaders, Sierra Leone, December 2014

Variable	Total no. (%) of respondents (*n* = 3540)^a^		No. (%) exposed to safe burial messages from religious leaders		*P*^b^
			Exposed (*n* = 3148)	Unexposed (*n* = 369)	
**Region**					
Western	812 (23)		667 (21)	135 (36)	< 0.001
Southern	562 (16)		512 (17)	45 (12)	
Eastern	919 (26)		824 (26)	95 (26)	
Northern	1247 (35)		1145 (36)	94 (26)	
**Sex**					
Male	1809 (51)		1621 (51)	175 (47)	0.14
Female	1731 (49)		1527 (49)	194 (53)	
**Age, years**					
15–24	1177 (33)		1031 (33)	136 (37)	0.11
≥ 25	2362 (67)		2117 (67)	233 (63)	
**Educational level**					
None	1194 (34)		1053 (33)	133 (36)	0.13
Primary	677 (19)		592 (19)	80 (22)	
Secondary or above	1668 (47)		1502 (48)	156 (42)	
**Religion**					
Islam	2335 (66)		2091 (66)	228 (62)	< 0.001
Christianity	1200 (34)		1054 (33)	139 (38)	
**Behavioural intentions**					
Intending to accept safe alternatives to traditional burials					
Yes	3049 (86)		2755 (88)	276 (77)	< 0.001
No	436 (13)		351 (11)	82 (23)	
Intending to avoid touching or washing the corpse					
Yes	3362 (95)		2992 (95)	347 (95)	0.57
No	170 (5)		150 (5)	20 (5)	
Intending to wait for the burial team to arrive					
≥ 2 days	1670 (47)		1548 (49)	115 (36)	< 0.001
< 2 days	1542 (44)		1328 (42)	203 (64)	
**Protective behaviours**					
Avoiding unsafe traditional burials					
Yes	1673 (47)		1538 (49)	127 (34)	< 0.001
No	1867 (53)		1610 (51)	242 (66)	
Avoiding physical contact with suspected Ebola patients					
Yes	1538 (43)		1421 (45)	110 (30)	< 0.001
No	2002 (57)		1727 (55)	259 (70)	

^a^ Column totals may not equal 100% of total sample size due to missing values (< 5%) for individual variables.

^b^ *P* values were determined from χ^2^ tests.

Note: Missing values for demographic variables represent < 1% of all responses: 1 missing for age, 1 missing for education, 3 missing for religion, 23 missing for exposure to religious messages. See data repository^26^ for missing values for behavioural intentions and protective behaviours.

A total of 3148 respondents (89%) reported that they had heard faith-based messages from religious leaders on safe Ebola burials and 369 (10%) had not heard these messages (data were missing for 23 survey respondents).

### Acceptance of safe practices

Overall, 3049 respondents (86%) intended to accept safe alternatives to traditional burials if a family member died during the Ebola outbreak. Exposure to religious leaders’ messages was positively associated with accepting a safe alternative to traditional burials (adjusted odds ratio, aOR: 1.69; 95% confidence interval, CI: 1.23–2.31; Table 2). The odds of reporting such intentions were three times greater among respondents from the southern region compared with the western region (aOR: 3.36; 95% CI: 1.46–7.70).

**Table 2:** Respondents’ intentions towards safe Ebola burials, by demographic characteristics and exposure to messages on safe burials from religious leaders, Sierra Leone, 2014

Variable	Intending to accept safe alternatives to traditional burials				Intending to avoid touching or washing corpse				Intending to wait up to ≥ 2 days for burial team to arrive		
	Yes	No	aOR (95% CI)		Yes	No	aOR (95% CI)		Yes	No	aOR (95% CI)
**Exposed to religious messages**											
No	276 (77)	82 (23)	1.0 (Ref.)		347 (95)	20 (5)	1.0 (Ref.)		115 (36)	203 (64)	1.0 (Ref.)
Yes	2 755 (89)	351 (11)	1.69 (1.23–2.31)		2992 (95)	150 (5)	0.89 (0.53–1.48)		1548 (54)	1328 (46)	1.84 (1.38–2.44)
**Age, years**											
15–24	1002 (86)	160 (14)	1.0 (Ref.)		1117 (95)	56 (5)	1.0 (Ref.)		533 (50)	538 (50)	1.0 (Ref.)
≥ 25	2047 (88)	276 (12)	1.16 (0.91–1.49)		2245 (95)	114 (5)	0.98 (0.68–1.42)		1137 (53)	1004 (47)	1.14 (0.95–1.37)
**Sex**											
Male	1564 (88)	220 (12)	1.0 (Ref.)		1723 (96)	82 (4)	1.0 (Ref.)		851 (52)	790 (48)	1.0 (Ref.)
Female	1485 (87)	216 (13)	1.03 (0.82–1.29)		1639 (95)	88 (5)	1.16 (0.84–1.60)		819 (52)	752 (48)	1.09 (0.92–1.28)
**Educational level**											
None	1040 (88)	139 (12)	1.0 (Ref.)		1123 (94)	67 (6)	1.0 (Ref.)		534 (51)	512 (49)	1.0 (Ref.)
Primary	587 (88)	81 (12)	1.05 (0.74–1.47)		643 (95)	32 (5)	0.84 (0.53–1.35)		322 (52)	293 (48)	1.25 (0.97–1.60)
Secondary or above	1421 (87)	216 (13)	0.92 (0.68–1.23)		1595 (96)	71 (4)	0.88 (0.59–1.31)		813 (53)	737 (47)	1.04 (0.84–1.29)
**Religion**											
Islam	2036 (89)	263 (11)	1.0 (Ref.)		2211 (95)	117 (5)	1.0 (Ref.)		1135 (54)	974 (46)	1.0 (Ref.)
Christianity	1010 (85)	172 (15)	0.98 (0.75–1.28)		1147 (96)	52 (4)	0.99 (0.68–1.45)		533 (49)	565 (51)	0.81 (0.67–0.98)
**Region**											
Western	648 (82)	146 (18)	1.0 (Ref.)		783 (96)	29 (4)	1.0 (Ref.)		427 (55)	354 (45)	1.0 (Ref.)
Southern	502 (92)	41 (8)	3.36 (1.46–7.70)		546 (97)	15 (3)	0.72 (0.32–1.62)		266 (53)	237 (47)	0.92 (0.45–1.91)
Eastern	791 (86)	123 (14)	1.80 (0.89–3.60)		869 (95)	48 (5)	1.44 (0.75–2.78)		378 (44)	487 (56)	0.50 (0.23–1.08)
Northern	1108 (90)	126 (10)	1.83 (0.95–3.52)		1164 (94)	78 (6)	1.78 (0.95–3.31)		599 (56)	464 (44)	0.92 (0.45–1.91)

aOR: adjusted odds ratio; CI: confidence interval; (Ref.): reference category.

Notes: Yes and No are the unweighted numbers (%) of respondents. We adjusted ORs for age, sex, education, religion and region. Total numbers of respondents for each variable may not exactly equal those in Table 1 due to missing data for behavioural intention and behaviour variables. Missing values for these variables are available in the data repository.^26^

Nearly all respondents (3049; 86%) intended to avoid touching or washing the corpse of a family member, regardless of exposure to religious leaders’ messages (aOR: 0.89; 95% CI: 0.53–1.48). None of the demographic variables were significantly associated with this outcome.

Half of the respondents (1670; 47%) intended to wait up to 2 or more days for the burial team’s arrival if they had a family member who was suspected of having Ebola. The odds of expressing this intention were almost twofold greater among respondents who were exposed to faith-based messages (aOR: 1.84; 95% CI: 1.38–2.44). Demographically, compared with respondents who self-identified as Muslims, those who self-identified as Christians had nearly a 20% decrease in their odds of intending to wait at least 2 days for the burial teams to arrive (aOR: 0.81; 95% CI: 0.67–0.98).

### Protective behaviours

When asked unprompted about their protective behaviours against Ebola, 1673 (47%) of the respondents cited that they were avoiding unsafe traditional burials. Exposure to religious leaders’ messages was associated with avoiding unsafe traditional burials among respondents exposed to faith-based messages compared with those who did not receive messages (aOR: 1.46; 95% CI: 1.14–1.89; Table 3). This outcome was further associated with residing in the southern (aOR: 2.43; 95% CI: 1.30–4.53) or eastern regions compared with the western region (aOR: 2.01; 95% CI: 1.21–3.50).

**Table 3:** Respondents’ reported protective behaviours, by demographic characteristics and exposure to messages on safe burials from religious leaders, Sierra Leone, 2014

Variable	Avoiding unsafe traditional burials				Avoiding physical contact with suspected Ebola patients		
	Yes	No	aOR (95% CI)		Yes	No	aOR (95% CI)
**Exposed to religious messages**							
No	127 (34)	242 (66)	1.0 (Ref.)		110 (30)	259 (70)	1.0 (Ref.)
Yes	1538 (49)	1610 (51)	1.46 (1.14–1.89)		1421 (45)	1727 (55)	1.65 (1.27–2.13)
**Age, years**							
15–24	552 (47)	625 (53)	1.0 (Ref.)		521 (44)	656 (56)	1.0 (Ref.)
≥ 25	1120 (47)	1242 (53)	1.08 (0.92–1.28)		1016 (43)	1346 (57)	1.10 (0.94–1.30)
**Sex**							
Male	854 (47)	955 (53)	1.0 (Ref.)		778 (43)	1031 (57)	1.0 (Ref.)
Female	819 (47)	912 (53)	0.97 (0.83–1.12)		760 (44)	971 (56)	1.11 (0.96–1.29)
**Educational level**							
None	538 (45)	656 (55)	1.0 (Ref.)		460 (39)	734 (61)	1.0 (Ref.)
Primary	3220 (47)	357 (53)	1.07 (0.86–1.34)		290 (43)	387 (57)	1.25 (1.00–1.55)
Secondary or above	815 (49)	853 (51)	1.13 (0.93–1.37)		787 (47)	881 (53)	1.56 (1.29–1.89)
**Religion**							
Islam	1112 (48)	1223 (52)	1.0 (Ref.)		1007 (43)	1328 (57)	1.0 (Ref.)
Christianity	560 (47)	640 (53)	0.98 (0.82–1.17)		529 (44)	671 (56)	1.02 (0.86–1.22)
**Region**							
Western	300 (37)	512 (63)	1.0 (Ref.)		293 (36)	519 (64)	1.0 (Ref.)
Southern	326 (58)	236 (42)	2.43 (1.30–4.53)		262 (47)	300 (53)	1.59 (0.91–2.79)
Eastern	490 (53)	429 (47)	2.01 (1.15–3.50)		443 (48)	476 (52)	1.84 (1.13–3.03)
Northern	557 (45)	690 (55)	1.29 (0.76–2.19)		540 (43)	707 (57)	1.48 (0.92–2.37)

aOR: adjusted odds ratio; CI: confidence interval; (Ref.): reference category.

Notes: Yes and No are the unweighted numbers (%) of respondents. We adjusted ORs for age, sex, education, religion and region. Total numbers of respondents for each variable may not exactly equal those in Table 1 due to missing data for behavioural intention and behaviour variables. Missing values for these variables are available in the data repository.^26^

When asked unprompted, 1538 (43%) respondents were avoiding physical contact with suspected Ebola patients to prevent Ebola transmission. Exposure to religious leaders’ messages was associated with avoiding Ebola patients (aOR: 1.65; 95% CI: 1.27–2.13). Residing in the eastern region was also associated with this outcome (aOR: 1.84; 95% CI: 1.13–3.03) compared with residing in the western region. Respondents with primary and secondary education had greater odds of avoiding Ebola patients when compared with those without any education (aOR:1.25; 95% CI: 1.00–1.55 and aOR: 1.56; 95% CI: 1.29–1.89, respectively).

### Intention to wait

Of those who intended to wait 2 or more days for burial teams’ arrival, 1670 were more likely to avoid unsafe burials compared with those 1542 who did not intend to wait that long (aOR: 1.49; 95% CI: 1.25–1.77; Table 4). Respondents from the southern and eastern regions had greater odds of avoiding unsafe burials compared with those from the western region (aOR: 2.92; 95% CI: 1.56–5.48 and aOR: 2.24; 95% CI: 1.28–3.93, respectively).

**Table 4:** Respondents’ reported protective behaviours, by demographic characteristics and intention to wait for burial team, Sierra Leone, 2014

Variable	Avoiding unsafe traditional burials				Avoiding physical contact with suspected Ebola patients		
	Yes	No	aOR (95% CI)		Yes	No	aOR (95% CI)
**Intending to wait for burial team to arrive**							
< 2 days	667 (43)	875 (57)	1.0 (Ref.)		620 (40)	922 (60)	1.0 (Ref.)
≥ 2 days	898 (54)	772 (46)	1.49 (1.25–1.77)		811 (49)	859 (51)	1.38 (1.17–1.63)
**Age**							
15–24	552 (47)	625 (53)	1.0 (Ref.)		521 (44)	656 (56)	1.0 (Ref.)
≥ 25	1120 (47)	1242 (53)	1.10 (0.92–1.31)		1016 (43)	1346 (57)	1.10 (0.93–1.31)
**Sex**							
Male	854 (47)	955 (53)	1.0 (Ref.)		778 (43)	1031 (57)	1.0 (Ref.)
Female	819 (47)	912 (53)	0.95 (0.81–1.11)		760 (44)	971 (56)	1.11 (0.96–1.29)
**Educational level**							
None	538 (45)	656 (55)	1.0 (Ref.)		460 (39)	734 (61)	1.0 (Ref.)
Primary	3220 (47)	357 (53)	1.01 (0.80–1.28)		290 (43)	387 (57)	1.23 (0.98–1.55)
Secondary or above	815 (49)	853 (51)	1.11 (0.91–1.36)		787 (47)	881 (53)	1.52 (1.25–1.86)
**Religion**							
Islam	1112 (48)	1223 (52)	1.0 (Ref.)		1007 (43)	1328 (57)	1.0 (Ref.)
Christianity	560 (47)	640 (53)	1.00 (0.83–1.20)		529 (44)	671 (56)	1.05 (0.88–1.26)
**Region**							
Western	300 (37)	512 (63)	1.0 (Ref.)		293 (36)	519 (64)	1.0 (Ref.)
Southern	326 (58)	236 (42)	2.92 (1.56–5.48)		262 (47)	300 (53)	1.81 (1.01–3.25)
Eastern	490 (53)	429 (47)	2.24 (1.28–3.93)		443 (48)	476 (52)	1.91 (1.13–3.22)
Northern	557 (45)	690 (55)	1.44 (0.84–2.45)		540 (43)	707 (57)	1.68 (1.02–2.75)

aOR: adjusted odds ratio; CI: confidence interval; (Ref.): reference category.

Notes: Yes and No are the unweighted numbers (%) of respondents. We adjusted ORs for age, sex, education, religion and region. Total numbers of respondents for each variable may not exactly equal those in Table 1 due to missing data for behavioural intention and behaviour variables. Missing values for these variables are available in the data repository.^26^

Intention to wait for over 2 days for burial teams was also associated with an increased avoidance of contact with suspected Ebola patients (aOR: 1.38; 95% CI: 1.17–1.63). Compared with the western region, residing in any of the three provincial regions was associated with avoiding Ebola patients: southern (aOR: 1.81; 95% CI: 1.01–3.25); eastern (aOR: 1.91; 95% CI: 1.13–3.22); northern (aOR: 1.68; 95% CI: 1.02–2.75). Having secondary education or higher was also associated with this outcome (aOR: 1.52; 95% CI: 1.25–1.86).

## Discussion

The results from this nationwide household survey conducted around the peak of the Ebola outbreak in Sierra Leone suggest that religious leaders may have been effective risk communicators during the outbreak response. Exposure to messages about safe burials from religious leaders was significantly associated with intending to adhere to safe burial measures, including accepting safe alternatives to traditional burials and intending to wait 2 or more days for a burial team to bury the corpse. Behaviourally, exposure to religious leaders’ messages was significantly associated with engaging in protective behaviours such as avoiding unsafe traditional burials and avoiding physical contact with suspected Ebola patients. Those who intended to wait for a burial team were also more likely to avoid unsafe traditional burials and physical contact with suspected Ebola patients.

Unsafe traditional burials played a key role in the transmission of Ebola during the outbreak.^9^^,^^11^ We found that the protective behaviour of avoiding unsafe traditional burials was more pronounced in the southern and eastern regions where lower numbers of cumulative Ebola cases were recorded compared with the western and northern regions that witnessed higher numbers of new cases around the time of the survey.^27^ Epidemiological data show that the eastern and southern regions were the epicentre of the outbreak in Sierra Leone.^11^ Therefore, the experience of living in the initial outbreak regions may have intrinsically reinforced protective behaviours in those regions through social learning.^28^

Intention to wait for 2 or more days for a burial team’s arrival may have been essential in minimizing the risk of unsafe burials. While government policy stated that all burial teams must respond to deaths within 24 hours, in practice they were sometimes unable to respond within this time frame.^18^^,^^20^ Our results show that exposure to messages from religious leaders was associated with the intention to wait for burial teams. Those who intended to wait for 2 or more days for the burial team to arrive had greater odds of self-reported avoidance of unsafe traditional burials. This finding is in line with the theory of planned behaviour, which emphasizes the important role that behavioural intentions may have on behaviour adoption.^29^^,^^30^ Therefore, an increased willingness to wait for burial teams and not performing an unsafe burial may have helped to reduce the spread of infection.

In our study, 10% of respondents reported they were not exposed to religious leaders’ messages at the time of the survey. This relatively small comparison group likely reflects the overall success of the religious leader’s communication campaigns during the outbreak. Apart from communicating Ebola messages through their preaching in mosques and churches, religious leaders were also involved in various media campaigns on radio and television to communicate the Ebola message during the outbreak.^12^

Trust is an essential component of effective risk communication during an outbreak and is likely one of the reasons why religious leaders were effective risk communicators during the Ebola outbreak.^12^^,^^21^ The Ebola messages were developed in line with public health advice but were tailored to suit the public need and delivered in a relatable way through scriptural texts. The respect that religious leaders gain from their religious communities nationwide provides a relatability to the messages they communicated.^7^^,^^31^^,^^32^ This public trust may enable religious leaders, as well as other community leaders, to be effective risk communicators during health emergencies.^33^

Limitations to this study include the potential for participants to provide socially desirable responses regarding their intentions and behaviours, especially given that the survey was conducted after intensified social mobilization efforts around the peak of the outbreak in the country. Due to the cross-sectional nature of the data, we cannot establish causality with the available data. Exposure to multiple information sources during the outbreak may also have made it difficult for respondents to discern the source of messages. Therefore, exposure to faith-based messages could have been conflated with exposure to other sources of information on Ebola burial prevention. To account for this, our primary independent variable was a question from the knowledge, attitudes and practices survey which was specific to religious leaders. A strength of this study is the large, national random sample obtained around the peak of the outbreak with a very high response rate. In the absence of quantifiable empirical data on the potential impact of religious leader engagements during any Ebola outbreak to date, our findings have important global public health significance by establishing a strong association between religious leaders’ messages and protective Ebola behavioural outcomes.

In conclusion, effective risk communication is a vital pillar in building a foundation of trust during health emergencies. Our results highlight the potential impact of religious leaders, who are trusted and respected figures in Sierra Leone, as effective risk communicators during the Ebola outbreak. Engaging religious leaders in risk communication in similar contexts may have a measurable impact in increasing protective behaviours that slow disease transmission during health emergencies. During the coronavirus disease-2019 (COVID-19) pandemic, effective risk communication and a cohesive interdisciplinary approach to health communication are essential. Protective behaviours such as physical distancing are again important components of the outbreak response.^34^ Lessons learnt from the 2014–2016 Ebola outbreak may provide valuable insights to optimize communication campaigns during the COVID-19 pandemic.
